# Association between the LDL/HDL ratio and sarcopenia in Chinese community-dwelling older adults

**DOI:** 10.1371/journal.pone.0339121

**Published:** 2025-12-16

**Authors:** Shanshan Lu, Cheng Wu, Yushuang Lin, Zhengkai Shen, Xiang Lu

**Affiliations:** 1 Department of Geriatrics, Sir Run Run Hospital, Nanjing Medical University, Nanjing, Jiangsu, China; 2 Department of Integrated Service and Management, Jiangsu Province Center for Disease Control and Prevention, Nanjing, Jiangsu, China; University of Ghana Medical School, GHANA

## Abstract

**Objectives:**

The link between lipid disorders and diverse diseases is amply documented. Yet, research probing how serum lipid levels tie in with sarcopenia remains scarce. This study delves into the connection between the LDL/HDL ratio and sarcopenia among elderly Chinese people.

**Methods:**

A total of 3,968 senior participants from Chinese communities were included in this cross-sectional study. To explore the relationship between the LDL/HDL ratio and sarcopenia, both a multivariate logistic regression model and a restricted cubic spline model were used. ROC curve analysis was employed to gauge how well the LDL/HDL ratio can detect sarcopenia.

**Results:**

Among the participants, 780 were diagnosed with sarcopenia. Multivariable logistic regression unveiled a significant positive correlation between the LDL/HDL ratio and sarcopenia. After adjusting for potential confounders, each unit increase in the LDL/HDL ratio corresponded to an approximately 3-fold higher odds of sarcopenia (OR = 3.01, 95% CI: 2.66–3.41, *P* < 0.001). A non – linear relationship between the LDL/HDL ratio and sarcopenia was confirmed by RSC analysis (*P* < 0.001). ROC curve analysis showed that the LDL/HDL ratio outperformed its individual components in predictive ability for sarcopenia.

**Conclusions:**

This study indicates that the LDL/HDL ratio may serve as an independent risk factor for sarcopenia.

## Introduction

The global population aging trend has fueled a rise in age – associated conditions [1]. Sarcopenia is characterized by a progressive deterioration in skeletal muscle mass, strength, and function with advancing age [2,3]. Its prevalence spans from 1% to 29% among older adults and may surpass 50% in institutionalized groups or those having comorbidities [4,5]. Studies show that muscle tissue declines at an annual rate of 2.1% starting at age 50, whereas muscle strength lessens by 1.5% yearly between 50 and 60 years old, and by up to 3% per year subsequently [6]. This age-related deterioration of skeletal muscle frequently co-occurs with other ailments like diabetes [7] and osteoporosis [8], and elevates the risk of falls, fractures, and a reduction in the capacity for performing daily living tasks [9].

With advancing age, adipose tissue undergoes a characteristic redistribution, a phenomenon that has been implicated in the pathogenesis of metabolic disturbances [10]. Disruption in lipid metabolism assumes a pivotal role in sarcopenia pathogenesis [11]. Fat infiltration into skeletal muscle can alter its structure, metabolism, and signaling pathways, thereby impairing muscle function and physical performance [12,13]. Therefore, it is essential to establish a reliable indicator to assess the relationship between sarcopenia and lipid levels. Traditional lipid biomarkers, including high-density lipoprotein cholesterol (HDL-C), low-density lipoprotein cholesterol (LDL-C), total cholesterol (TC), and triglycerides (TG), are constrained by limited specificity and sensitivity; consequently, their utility in elucidating lipid-mediated sarcopenia pathogenesis remains restricted [14,15].

The low-density lipoprotein cholesterol/high-density lipoprotein cholesterol (LDL-C/HDL-C) ratio has garnered significant attention as a marker indicative of lipid metabolism. LDL-C primarily transports cholesterol from the liver to peripheral tissues, and elevated levels are associated with increased risk of atherosclerotic plaque formation [16]. In contrast, HDL-C facilitates reverse cholesterol transport, removing excess cholesterol from peripheral tissues and atherosclerotic lesions for hepatic metabolism and excretion [17]. Recent research has shown a relationship between the LDL/HDL ratio, atherosclerosis, vascular stiffness [18,19], and cognitive function [20]. Additionally, it has been identified as a reliable predictor of cardiovascular events [21].

Nonetheless, the association between the LDL/HDL ratio and sarcopenia remains inadequately investigated, warranting further exploration. Elucidating these interrelationships may deepen understanding of musculoskeletal health maintenance in the aging population. The present study aims to investigate the associations between the LDL/HDL ratio and sarcopenia among elderly populations, with the intent of offering meaningful implications for subsequent clinical practice.

## Materials and methods

### Study participants

A cross-sectional study design was adopted, with a total of 3,968 participants enrolled. This study is part of a large-scale multicenter community research project initiated in 2020, within which the recruitment phase for the present sub-study began on 7 May 2020 and was fully completed on 28 October 2020. These were all healthy people who were undergoing routine health examinations in Yuetang Community,Yangzhou City, Jiangsu Province, China. The research protocol got ethical approval from the Institutional Review Board of Sir Run Run Hospital affiliated with Nanjing Medical University (approval number: 2019-SR-S041). All the procedures were in line with the Declaration of Helsinki as well as relevant national and institutional regulations. After fully explaining the study’s aims, procedures, potential risks and benefits, the right to withdraw without any negative consequences, and data-privacy safeguards to each participant, written informed consent was obtained from them. Data extraction and analysis for the present study were conducted in March 2025. Throughout this process, the research team did not have access to any participant-specific identifiable information.

Eligibility criteria for study inclusion were as follows: (1) participants were aged ≥ 60 years; (2) they exhibited autonomous movement capability to complete grip strength and gait speed assessments; (3) they provided voluntary consent for study participation. Exclusion criteria comprised: (1) presence of organic diseases (e.g., severe liver/kidney dysfunction); (2) malignant tumor diagnosis. Participant selection is outlined in S1 Fig.

### Data collection

In this study, participants underwent routine health examinations, completed sarcopenia-relevant assessments, and provided fasting blood samples for laboratory analyses. Grip strength was measured separately for both the left and right hands, with each hand tested three times consecutively and a 3-minute rest interval between measurements to avoid muscle fatigue. The blood analyses evaluated hemoglobin (Hb), fasting blood glucose (FBG), triglycerides (TG), total cholesterol (TC), alanine aminotransferase (ALT), aspartate aminotransferase (AST), low-density lipoprotein cholesterol (LDL-C), and high-density lipoprotein cholesterol (HDL-C), among other parameters. All biochemical assays were performed using the Siemens XPT Chemistry Analyzer. Body mass index (BMI) was calculated as weight/height², waist - to - hip ratio (WHR) as waist circumference/hip circumference, non-HDL cholesterol (Non-HDL-C) as TC-HDL-C, and remnant cholesterol (RC) as TC-HDL-C − LDL-C.

All procedures were executed by trained professionals possessing extensive expertise in their respective fields.

### Definitions of sarcopenia and LDL/HDL ratio

As per the Asian Working Group for Sarcopenia (AWGS) 2019 [3], sarcopenia is marked by reduced muscle mass, diminished muscle strength, and/or impaired physical function. Muscle mass in study participants was assessed via bioelectrical impedance analysis (BIA). Aligned with AWGS criteria, muscle mass is categorized as low when Appendicular Skeletal Muscle Index (ASMI) values fall below 7.0 kg/m² (males) or 5.7 kg/m² (females). Muscle strength was evaluated via grip strength measurements, with low – strength thresholds set at < 28 kg (males) and < 18 kg (females). Physical function was assessed using the 6 – minute walk test, where a walking speed < 1 m/s indicated reduced function. The LDL/HDL ratio was derived by computing LDL-C (mmol/L)-to-HDL-C (mmol/L) values [22].

### Statistical analysis

Numerical variables were analyzed per their distributional properties. Data deviating from a normal distribution were summarized via median and interquartile range, whereas categorical variables were described using counts and percentage ratios. The Mann – Whitney U test compared non – normally distributed data between two groups, and the chi – square test compared categorical variables. Participants were stratified into four groups based on LDL/HDL ratio quartiles: Q1, Q2, Q3, and Q4. A multivariate logistic regression model examined the association between the LDL/HDL ratio and sarcopenia.Restricted cubic splines (RCS) were employed to evaluate the potential non – linear association between them. The diagnostic ability of the LDL/HDL ratio in detecting sarcopenia was assessed through the receiver operating characteristic (ROC) curve. All statistical analyses were performed using R software (Version 4.4.1; R Foundation for Statistical Computing, Vienna, Austria), and a two-tailed *P* < 0.05 was considered statistically significant.

## Results

### Basic characteristics of participants

The characteristics of study participants are detailed in Table 1. A total of 780 sarcopenia cases were identified. Compared to non – sarcopenia individuals, those with sarcopenia were significantly older, with a higher male prevalence. Furthermore, sarcopenia patients exhibited significantly lower levels of BMI, WHR, educational attainment, marital status, FBG, Hb, TC, TG, ALT, ALT/AST ratio, HDL – C, RC, Non – HDL – C, ASMI, grip strength, and step speed (*p* < 0.05). In contrast, no significant difference in AST levels was observed between the sarcopenia and non-sarcopenia groups (*p* > 0.05).

**Table 1:** Characteristics of the participants.

Variances	Overall	Non-Sarcopenia	Sarcopenia	*p*
	n = 3968	n = 3188	n = 780	
**Age, y**	73.68 (70.51, 78.34)	73.00 (70.49, 76.97)	78.60 (73.49, 83.90)	<0.001
**Gender, n(%)**				<0.001
** Female**	1892 (47.7)	1404 (44.0)	488 (62.6)	
** Male**	2076 (52.3)	1784 (56.0)	292 (37.4)	
**BMI, kg/m** ^**2**^	24.56 (22.50, 26.74)	25.11 (23.24, 27.21)	21.95 (19.89, 23.77)	<0.001
**WHR**	0.91 (0.87, 0.94)	0.91 (0.87, 0.95)	0.90 (0.86, 0.93)	<0.001
**Educational level, *n* (%)**				<0.001
** No formal education**	1830 (46.1)	1419 (44.5)	411 (52.7)	
** Primary school**	1262 (31.8)	1037 (32.5)	225 (28.8)	
** Middle school**	678 (17.1)	575 (18.0)	103 (13.2)	
** High school or above**	198 (5.0)	157 (4.9)	41 (5.3)	
**Married, n (%)**				<0.001
** Yes**	3278 (82.6)	2697 (86.4)	581 (74.5)	
** No**	690 (17.4)	491 (15.4)	199 (25.5)	
**FBG, mmol/L**	5.44 (5.10, 5.97)	5.47 (5.12, 6.01)	5.32 (5.02, 5.76)	<0.001
**Hb, g/L**	129 (120, 138)	129 (121, 138)	127 (117, 136)	<0.001
**TC, mmol/L**	4.87 (4.30, 5.45)	4.89 (4.31, 5.46)	4.80 (4.24, 5.39)	0.025
**TG, mmol/L**	1.36 (1.00, 1.90)	1.41 (1.03, 1.97)	1.18 (0.92, 1.59)	<0.001
**ALT, U/L**	15.70 (11.40, 21.80)	16.30 (12.10, 22.72)	13.10 (9.40, 18.40)	<0.001
**AST, U/L**	23 (19, 28)	23 (19, 28)	23 (19, 28)	0.699
**ALT/AST**	0.68 (0.53, 0.87)	0.72 (0.56, 0.90)	0.55 (0.43, 0.71)	<0.001
**LDL-C, mmol/L**	2.01 (1.72, 2.36)	1.97 (1.65, 2.30)	2.25 (2.02, 2.58)	<0.001
**HDL-C, mmol/L**	1.41 (1.23, 1.60)	1.43 (1.26, 1.64)	1.27 (1.13, 1.38)	<0.001
**LDL/HDL**	1.46 (1.15, 1.76)	1.39 (1.08, 1.67)	1.78 (1.51, 2.15)	<0.001
**RC, mmol/L**	1.27 (1.08, 1.49)	1.28 (1.10, 1.51)	1.21 (1.03, 1.40)	<0.001
**Non-HDL-C, mmol/L**	3.40 (2.84, 3.96)	3.43 (2.88, 4.00)	3.22 (2.68, 3.74)	<0.001
**ASMI**	6.85 (6.14, 7.53)	7.13 (6.34, 7.71)	5.80 (5.30, 6.50)	<0.001
**Grip strength, kg**	24.00 (19.70, 29.50)	24.70 (20.50, 31.10)	20.70 (16.10, 24.90)	<0.001
**Step speed, m/s**	1.02 (0.91, 1.13)	1.04 (0.94, 1.14)	0.93 (0.79, 1.04)	<0.001

WHR, waist-to-hip ratio; BMI, body mass index; FBG, fasting blood glucose; Hb, hemoglobin; TC, total cholesterol; TG, triglycerides; ALT, alanine aminotransferase; AST, aspartate aminotransferase; ALT/AST, the ratio of ALT to AST; LDL-C, low-density lipoprotein cholesterol; HDL-C, high-density lipoprotein cholesterol; RC, remnant cholesterol; LDL*/*HDL-C ratio,low-density lipoprotein cholesterol/high-density lipoprotein cholesterol ratio; Non-HDL-C, non-HDL cholesterol.

### Association between LDL/HDL ratio and sarcopenia

A binary logistic regression analysis was conducted to assess the associations between LDL/HDL ratios and sarcopenia. As illustrated in Table 2, the risk of sarcopenia increases concomitantly with higher LDL/HDL ratios. These findings persisted even after adjusting for all confounding variables. In both unadjusted and fully adjusted models, the fourth quartile of LDL/HDL ratios was associated with significantly ORs of 4.87 (95% CI: 3.73–6.36; *p* < 0.001) and 18.19 (95% CI: 12.72–26.01; *p* < 0.001) for sarcopenia, respectively, when compared to the first quartile.

**Table 2:** Multivariable regression analysis of LDL/HDL quartiles in relation to sarcopenia.

Characteristic	Model 1		Model 2		Model 3	
	OR (95%CI)	*p*	OR (95%CI)	*p*	OR (95%CI)	*p*
**LDL/HDL ratio**	1.85 (1.70, 2.00)	<0.001	1.95 (1.79, 2.14)	<0.001	3.01 (2.66, 3.41)	<0.001
**LDL/HDL ratio quartile**						
** Q1**	1.000 (ref)		1.000 (ref)		1.000 (ref)	
** Q2**	2.75 (2.09, 3.63)	<0.001***	3.04 (2.26, 4.09)	<0.001***	4.92 (3.51, 6.89)	<0.001***
** Q3**	3.06 (2.33, 4.03)	<0.001***	3.59 (2.68, 4.82)	<0.001***	7.90 (5.59, 11.18)	<0.001***
** Q4**	4.87 (3.73, 6.36)	<0.001***	5.84 (4.39, 7.78)	<0.001***	18.19 (12.72, 26.01)	<0.001***
** *p* for trend**	<0.001***		<0.001***		<0.001***	

* p < 0.05; ** p < 0.01; *** p < 0.001.OR, odds ratio; 95% CI, 95% confidence interval;Ref, reference.

Model 1: No adjustment.

Model 2: Adjusted for age and sex.

Model 3: Fully adjusted model.

**Fig 1:**
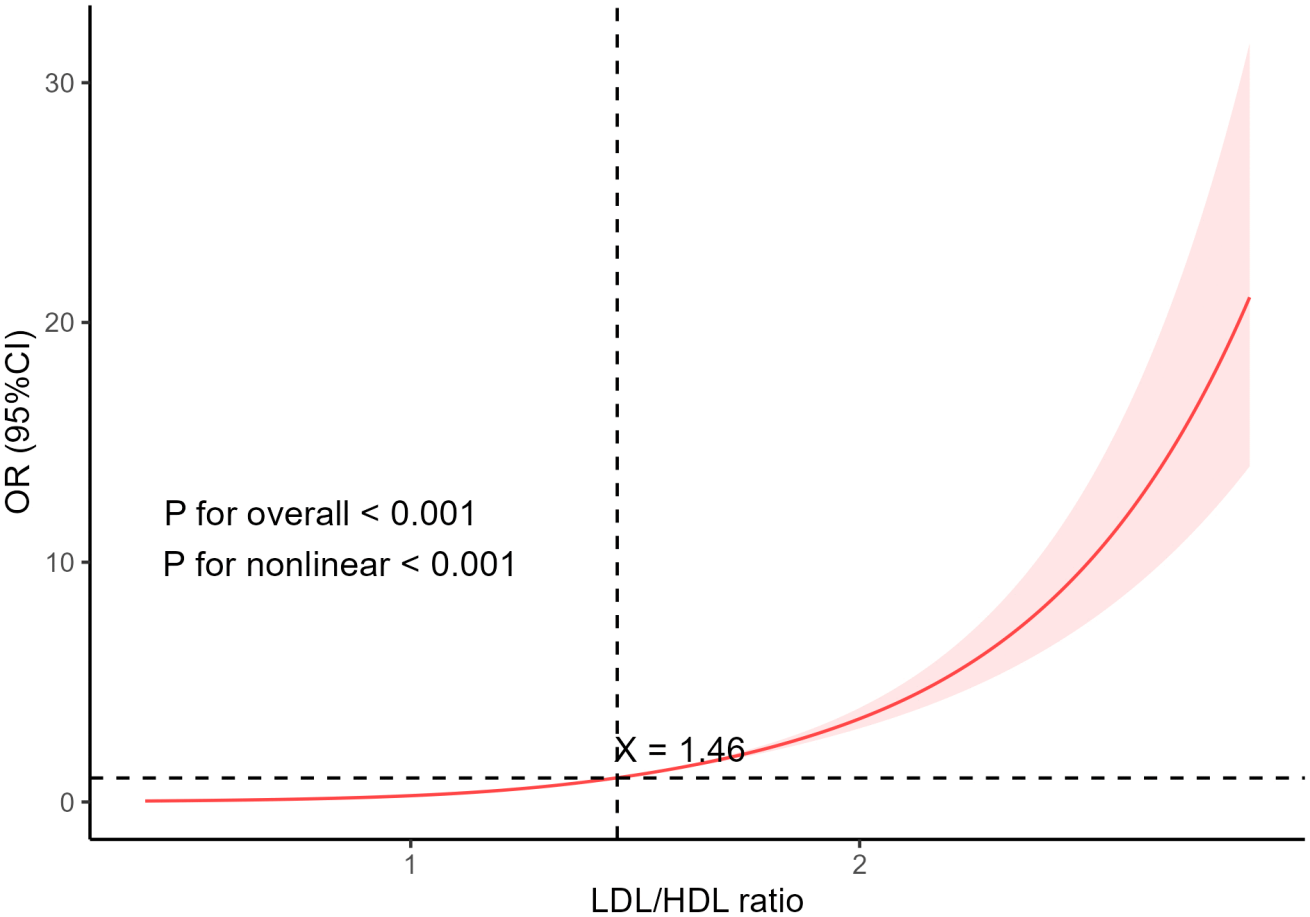
The dose-response relationship between the LDL/HDL ratio and the probability of sarcopenia occurrence.

Further investigations have corroborated the nonlinear relationship between LDL/HDL ratio and sarcopenia, as confirmed through restricted cubic spline (RCS) fitting, as shown in Fig 1 (*p* for non-linearity < 0.001).

### GAM analysis of LDL/HDL with ASMI, grip strength and walking speed

**Fig 2:**
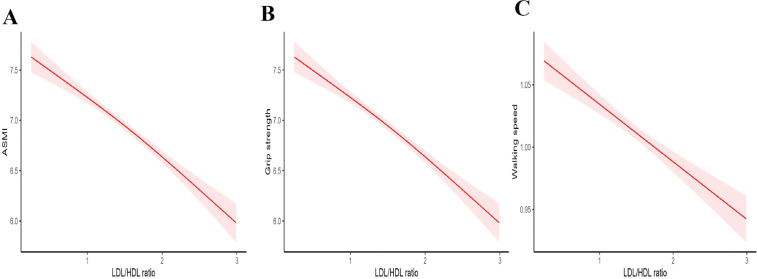
Correlation of the LDL/HDL ratio with ASMI, grip strength, and walking speed. (A) LDL/HDL ratio–ASMI correlation. (B) LDL/HDL ratio–grip strength correlation. (C) LDL/HDL ratio–walking speed correlation.

To further explore the LDL/HDL ratio–sarcopenia relationship, GAMs assessed associations of the LDL/HDL ratio with ASMI, grip strength, and walking speed. As shown in Fig 2A–C, a significant negative correlation was identified between the LDL/HDL ratio, ASMI, grip strength, and walking speed (*p* < 0.001).

### Subgroup analysis

**Fig 3:**
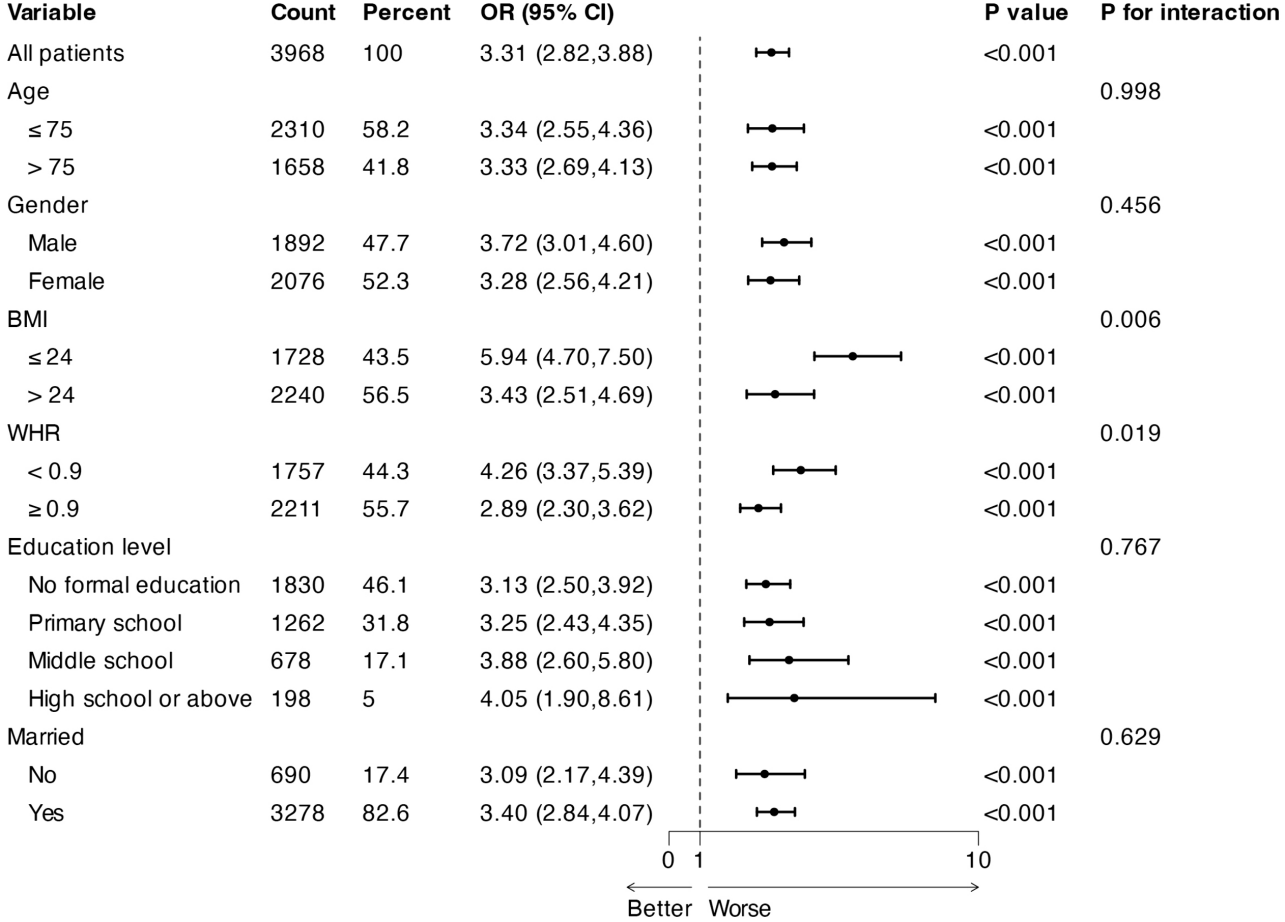
Subgroup analysis of the adjusted odds ratios for the LDL/HDL ratio and sarcopenia.

Analyses were stratified to evaluate possible modifications of the association between the LDL/HDL ratio and sarcopenia among different subgroups. Statistically significant interactions were detected in the subgroups after stratification by BMI and WHR (*P* for interaction 0.006 and 0.019), suggesting that the association between LDL/HDL ratio and sarcopenia might vary according to BMI and WHR (Fig 3). Nevertheless, no significant interactions were observed for age, gender, education level, or marital status, indicating the association may be unaffected by these factors (*P* for interaction >0.05).

### ROC curve assessment

**Fig 4:**
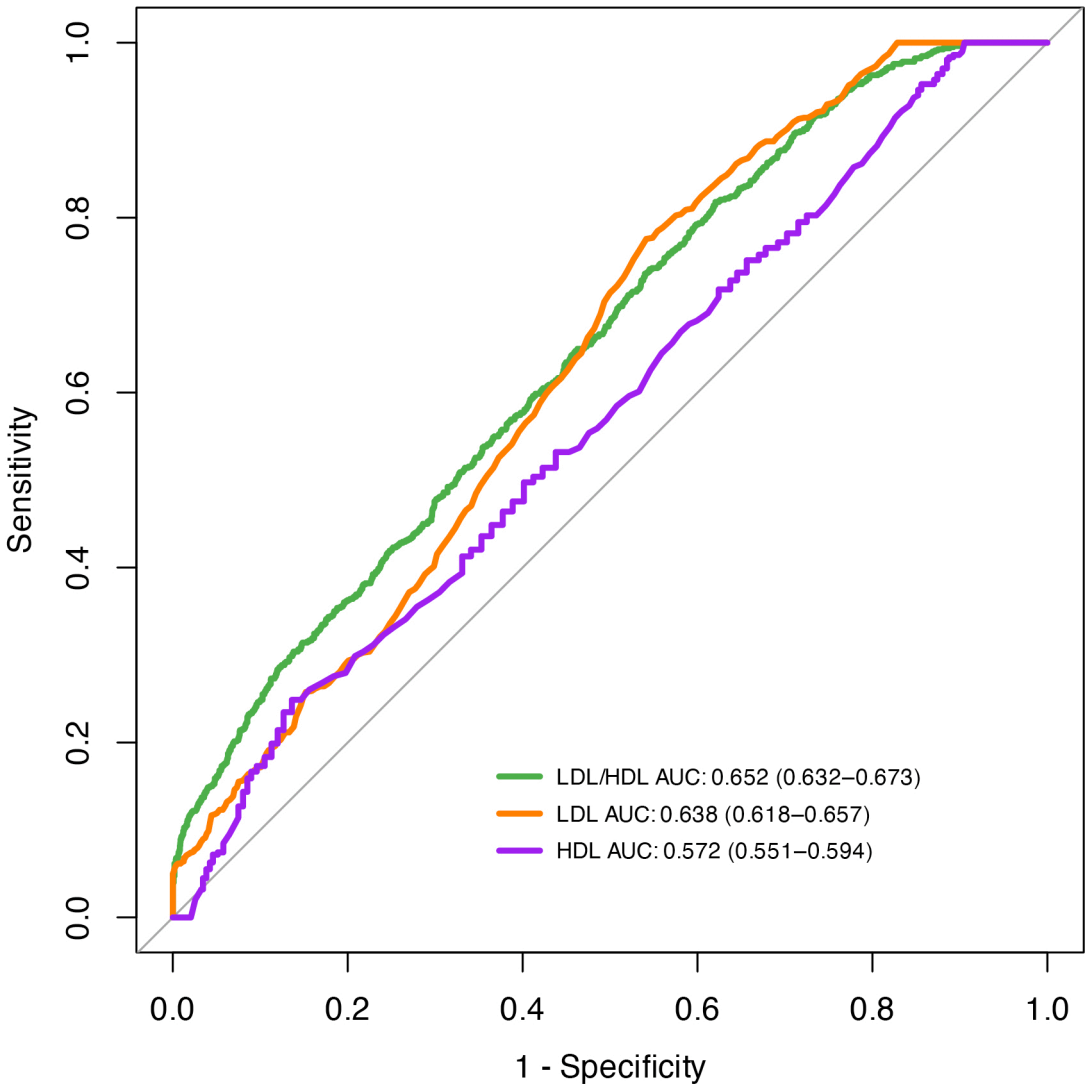
ROC curves for the association between LDL/HDL ratio sarcopenia.

To evaluate the LDL/HDL ratio’s diagnostic utility for sarcopenia, ROC curve analysis was performed (Fig 4). The AUC of the LDL/HDL ratio for sarcopenia diagnosis was 0.652 (95% CI: 0.632–0.673), versus 0.638 (95% CI: 0.618–0.657) for LDL-C and 0.572 (95% CI: 0.551–0.594) for HDL-C. These results indicate the LDL/HDL ratio outperforms either lipoprotein alone in diagnosing sarcopenia.

## Discussion

In this study, a notable positive correlation was identified between the LDL/HDL ratio and sarcopenia incidence among elderly Chinese individuals. Specifically, higher LDL/HDL ratios and ascending ratio quartiles were linked to a progressive increase in sarcopenia prevalence. This key finding was further validated by RCS analyses, which underscored the robustness of the dose-response relationship between the LDL/HDL ratio and sarcopenia risk.

Intermediates of lipid metabolism and fatty acids play crucial roles in maintaining skeletal muscle mass/function [23]. Age – associated inflammatory processes similarly affect lipid catabolism/release [24]. Within physiological limits, increased lipid metabolism – related parameters are protective against sarcopenia [25]. Aging involves increased adipose tissue deposition in skeletal muscle/bone marrow, contributing to muscle mass reductions [26,27].

Numerous studies report a significant correlation between HDL-C and LDL-C levels and sarcopenia incidence [28,29]. The present study’s findings align with these conclusions. These conventional lipoproteins are widely used in clinical settings for disease risk evaluation. Recent research has identified the LDL/HDL ratio as a more accessible, cost – effective lipid marker, offering greater predictive value for diabetes, cardiovascular/cerebrovascular diseases, and metabolic disorders vs. individual lipoproteins [30–32]. ROC analysis further assessed the predictive efficacy of HDL-C, LDL-C, and the LDL/HDL ratio for sarcopenia, revealing the LDL/HDL ratio outperformed LDL-C or HDL-C alone in predicting sarcopenia. However, it is important to acknowledge that the AUC of 0.652 for the LDL/HDL ratio remains suboptimal in clinical terms, and its predictive value may not be sufficient as a standalone diagnostic or screening tool for sarcopenia. This limitation could be attributed to the multifactorial nature of sarcopenia, which involves complex interactions between nutritional status, physical activity, hormonal factors, and other metabolic parameters not fully captured by this lipid ratio alone. Future prospective studies are needed to further validate the predictive value of the LDL/HDL ratio and enhance overall accuracy.

Subgroup analysis further elucidated variability in the LDL/HDL ratio and sarcopenia risk association across genders, age groups, and WHR. The association was particularly pronounced when considering BMI and WHR. These findings underscore the importance of focusing on these high-risk subgroups [33]. The underlying mechanisms may involve age-related fat redistribution in the elderly, hormonal fluctuations in women, and metabolic abnormalities [34–36]. BMI and WHR are established as reliable indicators of adiposity and obesity-associated metabolic dysfunction [37,38]. Importantly, obesity can trigger chronic inflammatory responses and exacerbate insulin resistance, both of which are implicated in the pathogenesis of muscle degradation [39,40].

Numerous previous studies have demonstrated an inverse correlation among grip strength, walking speed, and lipid levels [29,41]. Our findings are consistent with these studies, as GAMs indicated a significant negative correlation between the LDL/HDL ratio and appendicular ASMI, grip strength, and walking speed. This relationship may be attributed to lipid imbalances that impair muscle anabolism, compromise contraction function, and consequently affect muscle quality and strength [42].

This study suggests a high LDL/HDL ratio as an independent sarcopenia risk factor, though the exact mechanisms await elucidation. The findings indicate that, in comparison to traditional lipid measures, the LDL/HDL ratio is the most effective alternative indicator for evaluating insulin resistance [43].This may result from lipid accumulation inducing mitochondrial dysfunction, activating inflammatory factors and promoting their hypersecretion [25,44]. TAdditionally, insulin resistance occurs in this process. These factors interact in a cycle to accelerate sarcopenia progression [25]. AStudies indicate HDL levels enhance skeletal muscle mitochondrial metabolic function [45]. These findings supporting the importance of lipid management in sarcopenia.

### Strengths and limitations

This study represents the inaugural investigation into the relationship between the LDL/HDL ratio and sarcopenia among elderly individuals residing in Chinese communities. Stratified analyses were conducted to examine this association across various subgroups.

Nonetheless, several limitations must be acknowledged. The study population was restricted to individuals undergoing health examinations in communities within Jiangsu Province, which may constrain the generalizability of the findings to other regions or demographic groups. Despite comprehensive adjustments for numerous confounders, the potential for residual confounding cannot be entirely dismissed. The study has identified the LDL/HDL ratio as a risk factor for sarcopenia, warranting further research to elucidate the specific mechanisms and intermediate factors involved. Finally, given the cross-sectional nature of this study, establishing a causal relationship between the variables under investigation is not feasible.

## Conclusions

Our study discovered a notable correlation between the LDL/HDL ratio and the risk of sarcopenia. This result indicates that the LDL/HDL ratio might be a useful biomarker for evaluating sarcopenia, highlighting the significance of tracking and regulating LDL/HDL ratio levels among the elderly population.

## Supporting information

S1 Fig Participant selection process. (TIF)
